# The Impact of Chemotherapy on Hepatitis B Antibody Titer in Patients with Hematological Malignancies

**DOI:** 10.4274/tjh.2013.0342

**Published:** 2015-08-01

**Authors:** Münci Yağcı, Elif Suyanı, Merih Kızıl Çakar

**Affiliations:** 1 Gazi University Faculty of Medicine, Department of Hematology, Ankara, Turkey

**Keywords:** hepatitis B, Resolved infection, Hepatitis B surface antibody, Hematological malignancy, Chemotherapy

## Abstract

**Objective:**

To investigate the influence of chemotherapy (CT) on HBsAb titer in patients receiving CT due to hematological malignancy.

**Materials and Methods:**

The data of 75 patients who received CT with the diagnosis of various hematological malignancies and who had serum HBsAb levels measured prior to and after the cessation of CT were evaluated retrospectively.

**Results:**

The median age of the patients was 52 years (range: 16-78) with 49 (65%) males and 26 (35%) females. Median HBsAb titer decreased significantly after CT compared to the pre-CT median HBsAb titer [68 (range: 0-1000) vs. 100 (range: 6.2-1000)] (p=0.001). In subgroup analysis, median HBsAb titer decreased significantly after CT in acute leukemia patients [110 (range: 6.2-1000) vs. 67.8 (range: 0-1000)] (p=0.003) and in patients receiving intensive CT [97.2 (range: 6.2-1000) vs. 71 (range: 0-1000)] (p=0.036). The decrease in median HBsAb titer was significant in male patients (p<0.001). HBsAb became negative after CT in 9 patients who were HBcAb-negative and had lower pre-CT HBsAb levels.

**Conclusion:**

HBsAb decreased after CT, especially in acute leukemia and male patients, and in patients receiving intensive CT.

## Abstract (tr)

**Amaç:**

Bu çalışmada, hematolojik malignite nedeniyle kemoterapi alan hastalarda kemoterapinin Anti HBs antikor titresi üzerine olan etkisinin incelenmesi amaçlanmıştır.

**Gereç ve Yöntemler:**

Çeşitli hematolojik malignite tanısı ile kemoterapi alan ve hem kemoterapi öncesi hem de kemoterapi tamamlandıktan sonraki Anti HBs antikor titresi mevcut olan toplan 75 hasta retrospektif olarak incelendi.

**Bulgular:**

Hastaların ortanca yaşı 52 (16-78) olup 49’u (%65) erkek ve 26’sı (%35) kadın idi. Kemoterapi sonrası ortanca Anti HBs antikor titresinde kemoterapi öncesine göre anlamlı düşme saptandı [68 (range: 0-1000) vs. 100 (range: 6,2-1000)] (p=0,001). Alt grup analizi yapıldığında, kemoterapi sonrası ortanca Anti HBs antikor titresindeki azalmanın özellikle akut lösemi hastalarında [110 (range: 6,2-1000) vs. 67.8 (range: 0-1000)] (p=0,003) ve yoğun kemoterapi alan hastalarda [97,2 (range: 6,2-1000) vs. 71 (range: 0-1000)] (p=0,036) olduğu gözlendi. Ayrıca ortanca Anti HBs antikor titresindeki azalma erkek cinsiyette daha belirgin idi (p<0,001). Kemoterapi öncesi Anti Hbs antikor titresi düşük olan ve yine Anti HBc antikoru negatif olan dokuz hastada kemoterapi sonrası Anti HBs antikoru negatifleşti.

**Sonuç:**

Anti HBs antikor titresinde kemoterapi sonrasında azalma görülmüştür ve bu azalma akut lösemi hastalarında, erkek hastalarda ve yoğun kemoterapi alan hastalarda daha belirgin olarak saptanmıştır.

## INTRODUCTION

Hepatitis B virus (HBV) infection, existing in one-third of the world’s population, is a major health problem [1,2]. With the widespread use of various cytotoxic chemotherapies, it has arisen as a considerable clinical problem in patients receiving chemotherapy (CT) treatment. CT-induced HBV reactivation in patients with hematological malignancies leads to interruption of the treatment or even to the death of the patient due to hepatic failure [1,3,4].

The frequency of CT-induced HBV reactivation in hepatitis B surface antigen (HBsAg)-carrier patients with hematological malignancies is approximately 50% [2,4,5]. However, cases of resolved HBV infection, defined as HBsAg-seronegative, hepatitis B surface antibody (HBsAb)-positive, and/or hepatitis B core antibody (HBcAb)-positive, also carry the risk of HBV reactivation during or after the cessation of CT [1,2,3,4]. The frequency of HBV reactivation in patients with resolved infection has been investigated mostly in the context of allogeneic hematopoietic stem cell transplantation (HSCT) and varies between 11.6% and 50% [6,7,8]. In patients with resolved infection, there is usually a gradual decline of the HBsAb titer followed by the appearance of HBV DNA and HBsAg later on, leading to HBV reactivation [9,10].

From this point of view, we aimed to investigate the influence of CT on HBsAb titer in patients receiving CT due to hematological malignancy.

## MATERIALS AND METHODS

The data of 949 patients who received CT with the diagnosis of various hematological malignancies at the Gazi University Faculty of Medicine, Department of Hematology, between January 1995 and January 2012 were reviewed retrospectively. Hepatitis B serology was studied as part of the routine screening program prior to CT in our clinic. Patient files were evaluated for age, diagnosis, type and number of CT cycles, and serum HBsAg, HBsAb, HBcAb, hepatitis B e antigen (HBeAg), and hepatitis B e antibody (HBeAb). The HBsAb titer was measured by enzyme-linked immunosorbent assay (ELISA) and the cut-off point was 10 IU/L. The patients who were HBsAg-negative and had serum HBsAb levels prior to and after the cessation of CT were included in the analysis. The study was approved by the local ethics committee.

### Statistical Analysis

Statistical evaluation was done using SPSS 15. Data are presented as numbers and percentages or medians and ranges, as appropriate. The chi-square test was used for evaluating categorical values and the Wilcoxon U test was used for continuous values. All p-values are 2-sided with statistical significance at the 0.05 alpha level.

## RESULTS

Patient characteristics are presented in Table 1. Seventy-five patients receiving CT with the diagnoses of non-Hodgkin lymphoma (n=18), Hodgkin lymphoma (n=9), chronic lymphocytic leukemia (n=6), multiple myeloma (n=11), and acute myeloid leukemia (n=31) were included in the analysis. The types of CT applied to the patients were very heterogeneous and were grouped as follows: alkylating agents and/or anthracycline-based CT (n=21), nucleoside analog-based CT (n=6), demethylating agent-based CT (n=3), immunomodulatory agent and/or proteasome inhibitor-based CT (n=7), rituximab-based CT (n=14), and acute leukemia/high-dose CT (n=24). None of the patients had received autologous stem cell transplantation. The median age of the patients was 52 years (range: 16-78), with 49 (65%) males and 26 (35%) females. The median time from last dose of CT to first record of post-CT HBsAb titer was 52 days (range: 4-1334). The median time between pre- and post-CT HBsAb titers was 232 days (range: 42-15.619). Hepatitis B serology of the patients was as follows: all patients were HBsAg-negative, 31 (46%) of the patients were HBcAb-positive (data available for 68/75 patients), all patients were HBeAg-negative (data available for 62/75 patients), and 17 (29%) patients were HBeAb-positive (data available for 16/75 patients).

Median HBsAb titer decreased significantly after CT compared to the pre-CT median HBsAb titer [100 (range: 6.2-1000) vs. 68 (range: 0-1000)] (p=0.001). In subgroup analysis, while the decrease in median HBsAb titer in the lymphoproliferative disease group had only a trend for significance [98.7 (range: 8.1-810) vs. 82.5 (range: 0-810)] (p=0.072), in acute leukemia patients the decrease in median HBsAb titer after CT was significant [110 (range: 6.2-1000) vs. 67.8 (range: 0-1000)] (p=0.003) (Table 2). The decrease in HBsAb titer was also significant in patients receiving intensive CT, which was applied mostly for acute leukemia patients (n=21) [97.2 (range: 6.2-1000) vs. 71 (range: 0-1000)] (p=0.036). HBsAb titer results before and after CT in other CT groups were as follows: alkylating agent and/or anthracycline-based CT, 142 (range: 20.4-577.6) vs. 75.4 (range: 0-805) (p=0.068); nucleoside analog-based CT, 60.7 (range: 12.8-810) vs. 49 (range: 10.9-810) (p=0.225); demethylating agent-based CT, 51.6 (range: 11.7-505.5) vs. 45.9 (range: 43-368.5) (p=0.593); immunomodulatory agent and/or proteasome inhibitor-based CT, 42 (range: 8.1-644.7) vs. 45.7 (range: 0-779) (p=0.593); rituximab-based CT, 152 (range: 11-810) vs. 103 (range: 0-553) (p=0.064) (Table 2).

In univariate analysis, the decrease in HBsAb titer was not affected by age, type of treatment, HBcAb positivity, HBeAb positivity, or time from last dose of CT to time of post-CT HBsAb titer in acute leukemia group (p>0.05).

When the HBsAb titer decrease was investigated according to sex, median HBsAb titer decreased significantly for males (p<0.001). After subgroup analysis according to the disease diagnosis (lymphoproliferative disease vs. acute leukemia), the decrease was significant in males in both disease groups (p=0.04 and p=0.001). Subgroup analysis according to treatment type was also done; the decrease was significant in male patients receiving high-dose CT (p=0.009) and close to significant in male patients receiving rituximab-based CT (p=0.051).

HBsAb became negative after CT in 9 patients. Nevertheless, we do not have information about HBV DNA status at that time. However, liver function tests were normal at that time (median aspartate transaminase and alanine transaminase levels were 26 and 33 IU/L, respectively). Those patients whose HBsAb became negative after CT were not different from the patients who were HBsAb-positive in terms of sex, disease diagnosis, type of treatment, median time from last dose of CT to first record of post-CT HBsAb titer, and median time between pre- and post-CT HBsAb titers (p>0.05). However, the patients who became HBsAb-negative were HBcAb-negative (with 7/9 available data; p=0.013) and had lower median pre-CT HBsAb titer [22.4 (range: 11-81.6) vs. 142 (range: 6.2-1000)] (p=0.001).

## DISCUSSION

HBV reactivation, which is frequently encountered in patients with a diagnosis of hematological malignancy, has been well studied in the era of HSCT and CT. Seropositivity, high viral load, male sex, young age, intensive CT, corticosteroids, anthracyclines, cyclophosphamide vincristine, fludarabine, and monoclonal antibodies have been defined as risk factors for HBV reactivation [1,2,3,4,7,11]. On the other hand, effects of CT on the course of HBsAb titer have not been investigated in detail in patients receiving CT due to hematological malignancies including lymphoma, chronic leukemia, acute leukemia, multiple myeloma, and myelodysplastic syndrome. Francisci et al. studied the effect of CT on HBsAb titer in 43 patients and found that HBsAb titer decreased after CT, but in none of the patients did the HBsAb titer decrease below 10 IU/L. However, that study did not denote the type of CT, nor did it cover the subgroup analysis of hematological malignancies [12]. We retrospectively analyzed the course of HBsAb titer in our patients receiving CT with the diagnosis of various hematological malignancies. Similar to the findings for HBV reactivation, male patients and those receiving intensive CT were found to be more vulnerable to the decrease in HBsAb titer after exposure to CT. We also found that HBsAb decreased significantly in patients with the diagnosis of acute leukemia.

Although HBV reactivation frequency reaches 50% in HBsAg carriers receiving cytotoxic CT, patients with resolved infection are also at risk of reactivation [1,2,4,6,10]. HBV reactivation has been reported in lymphoma and acute lymphoblastic leukemia patients who had resolved infections [13,14,15,16,17,18,19]. Even high HBsAb titers might not prevent HBV reactivation in these patients, especially with the use of either high-dose CT or rituximab [17,18,19]. In the present study, HBsAb decreased in all CT groups, including rituximab-based CT; however, the decrease was most significant in the intensive CT group.

In our study it was observed that HBsAb became negative in 9 patients; however, their HBV DNA status at that time is not known. On the other hand, those patients were HBcAb-negative, implying that they were immunized previously, and they also had lower pre-CT HBsAb titers. The antigenic stimulation caused by the vaccine is less powerful compared to exposure to the virus itself, which makes those patients more prone to acute hepatitis B infection than HBV reactivation.

The decrease in HBsAb titer was more prominent in males in this study. Male sex is a well-known risk factor for HBV reactivation [1,2,3,4,7]. Women have higher numbers of B cells and produce higher levels of antibodies than men, which might be an explanation for this difference [20,21].

The underlying mechanism in the decrease of HBsAb titer after CT is probably destruction of antibodies producing B lymphocytes by CT. After exposure to HBV, CD4+ T cells trigger activation of both cytotoxic T cells and B cells [22]. Although cytotoxic T cells are the major contributors of HBV clearance by destroying infected hepatocytes, antiviral antibody production also has a substantial role in the control of infection [22,23]. As a result, HBsAg disappears and HBsAb appears, which is defined as resolved infection [1,24]. Despite this resolution, it is now well known that HBV DNA continues to exist in the liver, peripheral mononuclear cells, and even bodily secretions in minute amounts in subjects who were exposed to HBV [1,4,24,25]. In patients with resolved infection, there is usually a gradual decline of the HBsAb titer followed by appearance of HBV DNA and HBsAg later on, leading to HBV reactivation during CT or after the cessation of CT [9,10]. Serum alanine transferase levels usually rise after the elevation of HBV DNA, which may lead to the reactivation being missed [4].

The limitations of this study are its retrospective nature, leading to the lack of information about HBV DNA status of the patients, and the fact that the types of CT and diseases were heterogeneous. However, to our knowledge, despite its limitations, this study is the first investigating the effect of CT on the course of HBsAb titer in detail.

In conclusion, resolved HBV infection with HBsAb positivity is not a guarantee against HBV reactivation in patients receiving CT, and HBsAb decreases after CT, especially in acute leukemia and male patients and in patients receiving intensive CT. Therefore, patients who are at greater risk for the disappearance of HBsAb need close monitorization for the decrease and ultimate disappearance of HBsAb, appearance of HBV DNA, and HBV reactivation.

## Figures and Tables

**Table 1:**
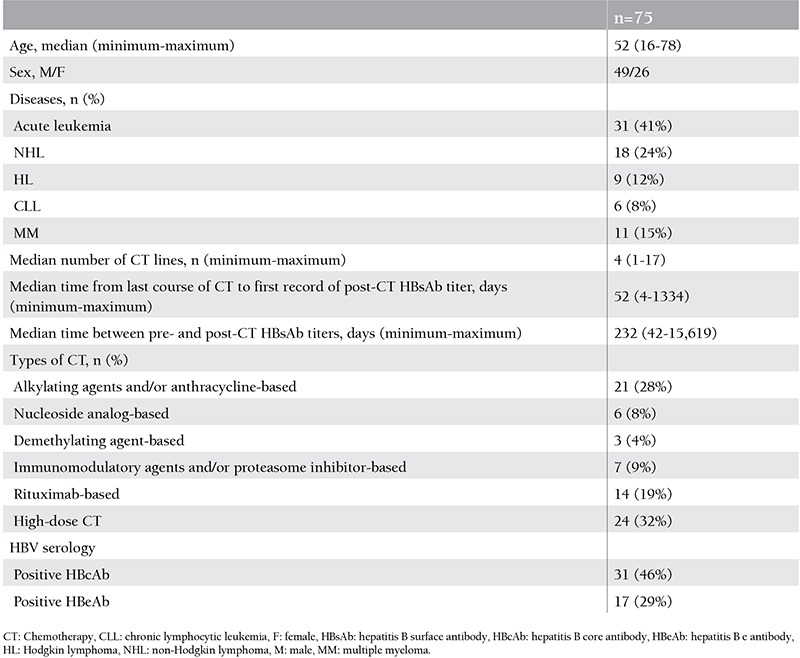
Patient characteristics.

**Table 2:**
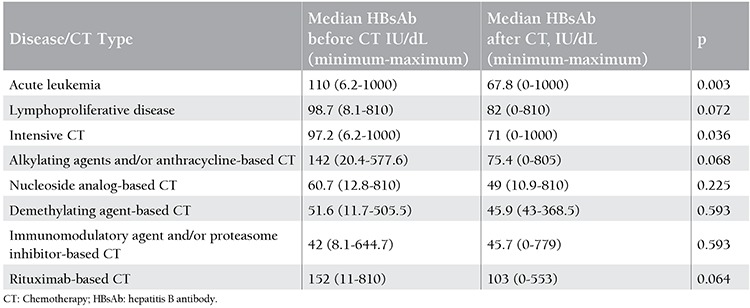
Median hepatitis B antibody levels prior to and after chemotherapy according to disease and chemotherapy types.

## References

[1] Yeo W, Johnson PJ (2006). Diagnosis, prevention and management of hepatitis B virus reactivation during anticancer therapy. Hepatology.

[2] Liang R (2009). How I treat and monitor viral hepatitis B infection in patients receiving intensive immunosuppressive therapies or undergoing hematopoietic stem cell transplantation. Blood.

[3] Firpi RJ, Nelson DR (2006). Viral hepatitis: manifestations and management strategy. Hematology Am Soc Hematol Educ Program.

[4] Lalazar G, Rund D, Shouval D (2007). Screening, prevention and treatment of viral hepatitis B reactivation in patients with haematological malignancies. Br J Haematol.

[5] Yağci M, Ozkurt ZN, Yeğin ZA, Aki Z, Sucak GT, Haznedar R (2010). Hepatitus B virus reactivation in HBV-DNA negative and positive patients with hematological malignancies. Hematology.

[6] Ramos CA, Saliba RM, Khorshid O, Shpall EJ, Giralt S, Patah PA, Hosing CM, Popat UR, Rondon G, Nieto Y, Champlin RE, Lima M (2010). Resolved hepatitis B virus infection is not associated with worse outcome after allogeneic hematopoietic stem cell transplantation. Biol Blood Marrow Transplant.

[7] Hammond SP, Borchelt AM, Ukomadu C, Ho VT, Baden LR, Marty FM (2009). Hepatitis B virus reactivation following allogeneic hematopoietic stem cell transplantation. Biol Blood Marrow Transplant.

[8] Knöll A, Boehm S, Hahn J, Holler E, Jilg W (2004). Reactivation of resolved hepatitis B virus infection after allogeneic haematopoietic stem cell transplantation. Bone Marrow Transplant.

[9] Knöll A, Boehm S, Hahn J, Holler E, Jilg W (2007). Long-term surveillance of haematopoietic stem cell recipients with resolved hepatitis B: high risk of viral reactivation even in a recipient with a vaccinated donor. J Viral Hepat.

[10] Firpi RJ, Nelson DR (2008). Management of viral hepatitis in hematologic malignancies. Blood Rev.

[11] Yağci M, Sucak GT, Haznedar R (2000). Fludarabine and risk of hepatitis B virus reactivation in chronic lymphocytic leukemia. Am J Hematol.

[12] Francisci D, Falcinelli F, Schiaroli E, Capponi M, Belfiori B, Flenghi L, Baldelli F (2010). Management of hepatitis B virus reactivation in patients with hematological malignancies treated with chemotherapy. Infection.

[13] Hui CK, Cheung WW, Zhang HY, Au WY, Yueng YH, Leung AY, Leung N, Luk JM, Lie AK, Kwong YL, Liang R, Lau GK (2006). Kinetics and risk of de novo hepatitis B infection in HBsAg-negative patients undergoing cytotoxic chemotherapy. Gastroenterology.

[14] Koo YX, Tay M, Teh YE, Teng D, Tan DS, Tan IB, Tai DW, Quek R, Tao M, Lim ST (2011). Risk of hepatitis B virus (HBV) reactivation in hepatitis B surface antigen negative/hepatitis B core antibody positive patients receiving rituximab-containing combination chemotherapy without routine antiviral prophylaxis. Ann Hematol.

[15] Sugauchi F, Tanaka Y, Kusumoto S, Matsuura K, Sugiyama M, Kurbanov F, Ueda R, Mizokami M (2011). Virological and clinical characteristics on reactivation of occult hepatitis B in patients with hematological malignancy. J Med Virol.

[16] Awerkiew S, Däumer M, Reiser M, Wend UC, Pfister H, Kaiser R, Willems WR, Gerlich WH (2007). Reactivation of an occult hepatitis B virus escape mutant in an anti-HBs positive, anti-HBc negative lymphoma patient. J Clin Virol.

[17] Sera T, Hiasa Y, Michitaka K, Konishi I, Matsuura K, Tokumoto Y, Matsuura B, Kajiwara T, Masumoto T, Horiike N, Onji M (2006). Anti-HBs-positive liver failure due to hepatitis B virus reactivation induced by rituximab. Intern Med.

[18] Sarrecchia C, Cappelli A, Aiello P (2005). HBV reactivation with fatal fulminating hepatitis during rituximab treatment in a subject negative for HBsAg and positive for HBsAb and HBcAb. J Infect Chemother.

[19] Ozaras R, Ar C, Ongoren S, Mete B, Tabak F, Mert A, Ozturk R (2010). Acute hepatitis B despite a previous high titer of anti-HBs. Hepatol Int.

[20] Oertelt-Prigione S (2012). The influence of sex and gender on the immune response. Autoimmun Rev.

[21] Abdullah M, Chai PS, Chong MY, Tohit ER, Ramasamy R, Pei CP, Vidyadaran S (2012). Gender effect on in vitro lymphocyte subset levels of healthy individuals. Cell Immunol.

[22] Bertoletti A, Gehring AJ (2006). The immune response during hepatitis B virus infection. J Gen Virol.

[23] Chisari FV (1997). Cytotoxic T cells and viral hepatitis. J Clin Invest.

[24] Yim HJ, Lok AS (2006). Natural history of chronic hepatitis B virus infection: what we knew in 1981 and what we know in 2005. Hepatology.

[25] Mason AL, Xu L, Guo L, Kuhns M, Perrillo RP (1998). Molecular basis for persistent hepatitis B virus infection in the liver after clearance of serum hepatitis B surface antigen. Hepatology.

